# Upholding justice through music: protesting betrayal in Oromo song, Wal Agarraa^☆^

**DOI:** 10.1016/j.heliyon.2022.e09956

**Published:** 2022-07-14

**Authors:** Kassaye Gutema Jebessa, Alemgena Belete Abdeta

**Affiliations:** Adama Science and Technology University, Department of English, Ethiopia

**Keywords:** Oromo, Political discourse, CDA, Protest music, Wal agarraa

## Abstract

The study aimed at explicating the linguistic choices subsumed in an Oromo protest song, Wal Agarraa by Galanaa Garoomsaa so as to shed light on the underlying messages as a form of political discourse. Music is considered one of the platforms for expressing discontent among the Oromo. In this research, we explored the textual instrumentality of the lyrics of the song as a form of political discourse in exposing the injustices perpetrated against the Oromo. We adopted Critical Discourse Analysis (CDA) framework to identify the discourses in the song. The study has made it evident that the song has got a number of underlying messages meant to raise the consciousness of the citizens regarding the injustices perpetrated against the Oromo. As such, the central theme of the song is that the Oromo quest for justice will be revealed eventually which the artist foreshadows using the title of the song **Wal Agarraa**/the time will come/. Furthermore, the functions of coercion, legitimization-delegitimization, and resistance, opposition and dissimulation have been subsumed in the song to convey the imbedded messages. The findings of the study also demonstrate that music goes far beyond simply reflecting and describing the state of affairs but it also becomes a platform through which discursive spaces are opened.

## 1 Introduction

Following years of Oromo Protests and resistance in many parts of the country, a promising political reform began in Ethiopia in 2018. However, the reform could not address the longstanding Oromo demands as the reform was hijacked by a reactionary and nostalgic group that had a plan of going back and resuscitating Imperial Ethiopia rather than moving forward and democratize the country. The coldblooded murder of a famous Oromo artist, Haacaaluu Hundessa, who played a pivotal role in popularizing the Oromo Protests, further aggravated the discontent among the Oromo. In the unrest that ensued following Haacaluu's murder, Oromos were murdered, and maimed in thousands. This was also followed by crack down on thousands of Oromos who are languishing in prisons including prominent Oromo politicians like Jaal Abdi Regassa and Colonel Gemechu Ayana. The murder of Haacaaluu remains mysterious to the dismay of the Oromo; justice has not been served yet. Thus, ‘**Wal Agarraa’** by a previous inmate of Haacaaluu was a work dedicated to portray what transpired during the reform pertaining to the lingering political demands of the Oromo and the murder of Haacaaluu.

Beyene (2019) underscored that the role of music as an instrument of political communication is evident in a predominantly oral society like Ethiopia, where music plays an important role in expressing discontent and exposing injustice. Similarly, the Oromo have got rich oral tradition. And music is an integral part of Oromos' rich oral forms of communication. Among the Oromo, protest songs are considered both a form of art and an ingredient of day-to-day activities. King and Richard (1995: 19) noted that music as a form of communication, “…speaks directly to society as a cultural form”. It can thus be drawn from this notion that different musical codes portray and configure different experiences of individuals and communities. Thus, music is considered as a platform which subsumes different aspects of people's lives and hence a form of communication. During the Oromo protest, popular songs have taken center stages as tools for popularizing the injustice facing the Oromo society.

Furthermore, Särkämo (2018) noted that music helps to persuasively communicate varieties of issues at different levels. These levels are at the cognitive, physical and emotional level. This implies that music can help convey different themes. Similarly, King and Richard (1995) opine that music does not only rouse the wish to dance but it also in the same vein invokes one to realize the social, political, and economic conditions at a given point in particular time.

What is more, music is a basic human function which provides a platform for people to express their joys, anguish and what they believe about a particular system. Music can also help to keep a record about people's history, travesties and trajectory. When events occur, artists compose music in the form of reaction. Therefore, music could be considered as a socially and politically functional tool. This paper does not focus on the melody, the harmony and the rhythm; it is rather based on the lyrical and thematic content of the selected song. This paper focuses on the analysis of an Oromo resistance song released in 2020 so as to identify and discuss the political, economic and social themes subsumed in it.

### 1.1 Objectives of the study

The major objective of the study was to discern the messages of Oromo protest song titled ‘**Wal Agrraa’** by Galanaa Gaaromsaa.

The specific purposes of this study were, therefore, to.

a) expound the underlying messages in the song as a form of political discourse
b) identify linguistic choices in the song titled ‘***Wal Agarraa’*** by Galanaaa Garomssaa;
c) explicate the linguistic choices in light of strategic functions of political discourse

## 2 Review of related literature

As it has been noted by different scholars, music serves to check the level of grudges felt by a given society. In connection with this, Ashworth (2016:5) noted, “Music gauges the temperature of society. Angry, despairing lyrics indicate a faction of society containing angry, despairing, and likely politically dissatisfied people.” It could be seen from this notion that music is a means to express political views and to initiate ordinary citizens to incite political change. Music is one of the platforms for expressing the situation of a given society at a particular point in time. As such it could be argued that music is an instrument for advocating political awareness to ordinary persons.

Music is an easy way to let oneself to be recognized as anyone who wants just to sing along and be part of the story that is being told. As a trend, people use music as a platform when they face injustices. Accordingly, people would sing against crimes and against inhumanity such as vandalism, brutality, racism, war-mongering and all other social ills that faced them (Akingbe and Onunaga, 2020). Musicians have often managed to mobilize the mass to realize social ills. They enable people to have a critical look at what is going on at a particular time and place. It has often been seen that various styles of music and songs are expressions of the society they originate from, directly or indirectly. As a result, music can never be completely isolated from the socio-political, socio-economic and cultural circumstances it is a part of.

According to Hansen (2007) protest music is something that objects to injustice, whether it is based on social, economic, political or racial circumstances. The protest musician attempts to raise the awareness of the subject so that they can take actions in reaction to injustices. Protest songs usually contain elements of subversion and controversy. They are traditionally considered to be folk music, or belong to folk music genres.

According to van Leeuwen (2012 cited in Akingbe and Onanuga (2020) music is considered as a discourse that could be scrutinized as it is a part of social, political and economic life. This assertion seems to correspond with our labelling of ‘Wal Agarraa’ as a political discourse. The scholars further assert that through music emotive allegiance can be formed as part of the effort to raise the awareness of the masses. Therefore, it is possible to conduct the linguistic analysis of the genres of music to illuminate its contents. In line with this assertion, it is necessary to consider the selected song as a discursive text. This implies that the social, cultural, political and economic perspectives of the discourse could be analysed within the context of the study.

Protest music is considered as subcategory of literature in which protest is the major theme (Akingbe, 2012). As such, this literature will comprise a categorical viewpoint and a vivid perception of what is at stake and an optimistic belief in the ultimate victory for justice. This notion implies that the major purpose of protest literature is to vividly reveal the issues that are at stake in societies and enable the society to realize the issues. The overall goal of protest literature is to help the community comprehend the challenges that are hindering them and thereby instil particular ideology in the people so that they can take collective actions. Thus, protest music instigates fighting injustice, mistreatment and discrimination of any sort facing a certain group or nation. In doing so, it raises the level of consciousness of a given community so that the community can reach a vantage point to realize the adverse situation and take some kind of action.

Several scholars have studied the role of music in fighting injustice in Ethiopia. Beyene (2019), Ogret (2008) and Adam and Mamphilly (2015) are the prominent ones among them. For instance Beyene (2019) has noted that the study of the relationship between music and politics in Ethiopia helps to discern the patterns and trends of injustice and social evils across different time intervals. According to this scholar, such insights will enable one to understand emerging and recurring challenges affecting the country and society at large. To this end, scrutinizing the role of protest songs pertaining to **Wal Agarraa** could shed light on the major social evils perpetrated in that time span.

The song by Galanaa Garomsaa, **Wal Agarraa**, appears to clearly portray the process of movement in Ethiopia due to the political instability in the country. The setting of the song extends to the glorious days the of Gadaa System and expresses the upheaval by Oromo youth, particularly the Oromo Protests. The song subsumes the glory, sense of resentment resulting from betrayal and the desire to reclaim lost pride and glory. The song serves as a tool to voice the issues of the Oromo in association with major incidents that have transpired in the post Oromo^1^ Protest period.

### 2.1 Theoretical framework

Chilton (1985) elaborated the relationship between language and politics by underscoring that politics cannot be conducted without language. Thus, it is the use of language in the constitution of social groups that leads to politics. In a similar vein, Pelinka (2007) stated that the study of language goes far beyond the scopes of literature and linguistics. The scholar further noted that ‘‘language must be seen (and analysed) as a political phenomenon’’ (2007:129). It is possible to draw from these notions that politics should be thought of as a discursive phenomenon which is worth scrutinizing.

The theoretical framework selected for analysing political discourse subsumed in **Wal Agarraa** is the one developed by Chilton (1985). We selected this model because of its comprehensiveness (Colima and Cabegas, 2017). This model is a model of political discourse analysis which proposes a linguistic analysis on three levels. The model has got pragmatic, semantic and syntactic levels which are directly related to the strategic functions characterizing the political discourse of dissimulation, legitimation and delegitimization, resistance, opposition and protest was used.

### 2.2 Methods of the study

This study was an undertaking meant to expound the nature and content of **Wal Agarraa**/we shall see each other/, an Oromo protest song which was very popular. The study was meant to explicate the textual analysis of the political instrumentality of the lyrics used in the selected song. We consider this song as a form of political discourse because, having experienced the Oromo Protest as Oromo first-hand and having witnessed the miseries and abuses of the protesters and the hijacking of the revolution, we have realized that the song was politically loaded and hence it reflected what transpired in due processes. The paper lends itself to the mixed method as auto-ethnographic method has been used along with descriptive and interpretive perspectives of qualitative approach for analyzing the linguistic devices used in the song. Auto-ethnography is a kind of research in which researchers conduct and write ethnographies of their own people (Hayano 1979). As such, we have aspired to expound the themes in **Wal Agarraa** from our lived experiences.

As it has been noted by Fairclough (1995) discourse creates and reproduces the social world, by using language. To this end, language that is used for describing a given situation is not impartial or innocent for it has a bearing on what is taking place in the different realms of life. Consequently, language is considered as a social construction. Fairclough also underscored that there is a dialectical relationship between language and discourse which implies that language influences the context in which it occurs and the context in turn, influences language production.

The specific descriptive and interpretive method used for analyzing the resistance song, ***Wal Agarraa****/the time will come/*, was critical discourse analysis (CDA). CDA as an approach combines some sort of textual (linguistic) theories and analysis with socio-political and critical theories and analysis. Luke (2002: 100) cited in Breeze (2011:495) noted that “CDA involves a principled and transparent shunting backwards and forth between the microanalysis of texts using varied tools of linguistic, semiotic and literary analysis, and the macro-analysis of social formations, institutions and power relations that these texts index and construct”. Accordingly, we analyzed the song by using various linguistic, semiotic and literary tools. Moreover, we have drawn the implications of the macro level analysis of institutions and power relations.

One of the tenets of CDA is that, it comprises two main traits which relate to how power and ideology operate in the public sphere. It envisions examining how language contributes to and maintains power and ideology. Consequently, most definitions of CDA will then focus on emphasizing the relationship between language and context. The discourse can be represented by a written text or a spoken word. In connection with this, the current study borrows Breeze's (2011:495) stance that, ‘‘CDA will therefore be used in an inclusive sense, to mean the broad body of theory and research generated by specialists who regard themselves as critical discourse analysts in one sense or another.’’ Thus, Fairclough's three-dimensional model has been used for making the analysis. In a nut shell the study is linguistic analysis of how linguistic features and stylistic choices have been employed in the representation of different contexts in the selected song.

### 2.3 The lyrics of Wal Agarraa

Galanaa Garomsa 2021 Wal Agarraa/the time will come/

[1] **Nan geerara aarii koo**/I will express my anger through ‘gerarsa’
[2] **kan na keessaa boba'u**/the fury that is burning in my heart
[3] **Yoo gaalee ta'e malee**/clay can only be pot;
[4] **Supheen gaachana hin ta'u**/as it can't be an armour
[5] **Leencca ta'e dhaladheen**/I was born as a lion
[6] **kodhoollemmoo ta'a ree**/how could I be a coward
[7**]namni bakakkaa beeku**/he who is accustomed to the sound of thunder
[8] **Dibbefimmo nannahaa** ree?/doesn't shake when drums are beaten
[9] **Ijoollee biyya kootii**/my fellow countrymen
[10] **Gugurraa mataan** furaa/black people with plaited hair
[11] **Eenyutu eenyuun haale**/who has betrayed whom?
[12] **Maal irbuun gaafa duraa**/what was the oath from the outset?
[13] **Osoo birbirsi jiru**/in the presence of ‘birbirsa’- podocarpus falcatus
[14] **Maaltu faarsa miciree**/no one will appreciate bushes
[15] **Guddaan gaanfa Afrikaa**/the giant of Horn of Africa
[16] **Oromookoo mitiiree?**/is it not my Oromo?
[17] **Oromiyaa mitiiree?**/is it not Oromia?
[18] **Sirna gadaa mitiiree?**/is it not Gadaa System?
[19] **Oromummaa mitiiree?**/is it not Oromumma?
[20] **Gadaa fi dhugaadha kallachi keenya** (x2)/Gadaa and truth are our emblem
[21] **Jifuu fi safuudha kabajni keenya** (x2)/norms and ethics are our principles
[22] **Gadaa malee hin faarsinu gadoo**/we appraise Gadaa; not misery
[23] **Lubaan bullaa hin qabnu madoo**/we want to be led by elites; not the wicked;
[24] **Nan gala hin jedhu namni osoo hin geenye**/no one will return before he's reached his destination
[25] **Fala hin argatu namni faloo hin beekne**/he who doesn't have wisdom can't get expiation
[26] **Na nyaartulle hinnawu gonkaa**/even if you frown at me; I won't be frightened
[27] **Dhugaa irraa maaltu na dhorkaa**/who can prohibit me from pursuing truth
[28] **Eessatti kufee namni dhugaaf gulee**/what has befallen to he who upheld truth
[29] **Galgalli hintoluu namni sobaaf duulee**/the destinies of those who pursue falsehood are futile
[30] **Egaa ergan kudhan obsee**/I tolerated some errors
[31] **Kudhan dhoqsee kudhan numan gorsee**/I hid some; I gave him council for some other errors
[32] **Hima didnaanan gubuuf itti dabsee**/when he refused my counsel, I tilted it to burn
[33] **Obsii daangaa darbu qabaa**/tolerance has got limits
[34] **Harkii bokkuu qabee bodees numa qabaa**/the same hand that holds bokku (symbol of power)can also bear a spear
[35] **Natu sii baataa siituu of dadhabaa**/I catered for you tirelessly but you despised my catering
[36] **Obsuufi dhoksuu koo dabuma seetee?**/I concealed your errors but you took my patience as cowardice/
[37] **Ni beektii garaan koo akka na gootee**/Instinctively I knew what you have done against me
[38] **Sibiilallee sibiilatuu qaraa**/it is only metal that can file knife
[39] **Dhugaan namaa galggala galaa**/justice will always be done eventually
[40] **Hadhaba cabsaa inni karaa beeku**/one who knows the way evades the risky places
[41] **Haqatti hingamuu namnii haqqii beekuu**/he who knows truth won't violate justice
[42] **Sangaan usnaan iyyite qacceen**/the whip shouted louder though the ox kept silent
[43] **Beekaa riphnaan dhaadatti dawween**/we hid our resentment; but the coward is threatening
[44] **Eenyutu na'aaf bubbutaree nyaara**/no one will startle when you frown
[45] **Keessi hin mul'atu beeki garaan dhiiraa**/no one knows what is there in a hero's heart
[46] **Maaf na nyaaraa na nyaaru kanarra**/why does he frown at me; he can't frown at me anymore
[47] **Maalan aarasi hin aaru kanarra**/why do I get angry; I won't be riled anymore
[48] **Maalan boo'a hin boo'u kanarra**/why do I weep; I won't weep anymore
[49] **Yoo jiraanne, birraa wal agarra** (x2)/we shall see each other in the spring if we could make it
[50] **Yoo jabaanne, birraa wal agarra**/if we could manage it, we shall see each other in the spring
[51] **Yoo wal taane, moonee wal agarra** (x2)/if we are united, we shall see each other after victory
[52] **Wal agarra garraa, Wal agarraa garraa** (x2)/we shall see each other; we shall see each other
[53] **Wal agarrees jirra** (x2)/we have seen each other
[54] **Walis arguuf jirra** (x2)/we are to see each other
[55] **Wal agarra!!**/we shall see each other
[56] **Birraa wal agarraa**/we shall see each other in the spring
[57] **Yaa ijoollee…yaa ijoollee…**/my people…my people
[58] **kan jenne hin oolle** (x2)/the one we speculated has materialised
[59] **Yaa ijoollee…yaa ijoollee…**/my fellows…my fellows (x2)
[60] **Ammas na foolee(**2)/once again it has beset me
[61] **Gadaa malee hin faarsinu gadoo**/we appraise Gadaa; not misery
[62] **Lubaan bullaa hin qabnu madoo**/we want to be led by elites; not the wicked;
[63] **Nan gala hin jedhu namni osoo hin geenye**/no one will return before he's reached his destination;
[64] **Fala hin argatu namni faloo hin beekne**/he who doesn't have wisdom can't get expiation
[65] **Nanyaartulle hinnawu gonkaa**/even if you frown at me; I won't be frightened
[66] **Dhugaa irraa maaltu na dhorkaa**/no one can prohibit me from pursuing truth
[67] **Eessatti kufee namni dhugaaf guulee**/what has befallen of he who upheld truth
[68] **Galgalli hintoluu namni sobaaf duulee**/the destinies of those who pursue falsehood are futile
[69] **Dhugaan qaxxisa tiratti**/truth moves slowly
[70] **Akka arge hineemtu karaadha filatti**/truth does not fan out; it travels on selected paths
[71] **Gaafa olbaatu tulluudha nimul'atti**/upon reaching the top it is observed like a hill
[72] **Gowwaan hosseen karra cufa**/the foolish shuts a door using hollow barks
[73] **Namni kufuu hawwe teessumatti kufa**/one that opts to fail would fall from the seat
[74] **Gara kuteessatti dukkanni ni ifa**/but to the brave, darkness will glow
[75] **Obsuufi dhoqsuu koo dabuma seetee?**/I covered up your errors but you contemned my effort
[76] **Ni beektii garaan kooakka na gootee**/I knew instinctively what you have done against
[77] **Sibiilallee sibiilatuu qaraa**/it is only iron that files knife
[78] **Dhugaan namaa galggala galaa**/justice will always be done eventually
[79] **Hadhaba cabsaa inni karaa beeku**/one who knows the way evades the risky places
[80] **Haqatti hingamuu/namnii haqqii beekuu**/he who knows truth won't violate justice
[81] **Sangaan usnaan iyyite qacceen**/the whip shout louder though the ox keeps silent
[82] **Beekaa riphnaan dhaadatti dawween**/even if we hide our resentment; the coward is threatening
[83] **Eenyutu naaaf bubbutaree nyaara**/no one will startle when you frown
[84] **Keessi hin mul'atu beeki garaan dhiiraa**/make no mistake; no one knows what is there in a hero's heart
[85] **Gaafa dhiiraa lubbuun haa dabartu**/let life be lost
[86] **Eessa abbaashii silas du'a hin haftu**/for death is inevitable
[87] **Gaafa giitii lubbuun haa dabartu**/on the eventful day may life be lost
[88] **Eega Haacaaluu lubbuun nan marartu**/I don't fancy life after Haacaaluu's death
[89] **Eega dhiiraa lubbuun haa dabartu**/let life be lost for a hero
[90] **Gaafa giitii lubbuun haadabartu**/let life be lost on an eventful day
[91] **Gaafa gootaa lubbuun nan marartu**/I don't hesitate to bet my life for the hero
[92] **Gaaf dhugaa kee lubbuun nan maratu**/I don't hesitate to lose life for your cause
[93] **Wal agarraa garraa** (2)/we shall see each other
[94] **Wal agarrees jirra**/we have already seen each other
[95] **Walis arguuf jirra**/we are yet to see each other
[96] **Birraa wal agarraa**/we shall see each in the spring

### 2.4 Analysis of the lyrics of Wal Agarraa

The units of analysis of the corpus of the study were the verses. Thus, we collected the corpus, transcribed the song lyrics and segmented them into verses. After carrying out thematic analysing of the song lyrics we analysed them in the three different linguistic levels viz: pragmatic, semantic and syntactic. In the pragmatic level, the speech acts and uses of pronouns were analysed; in the semantic level lexical fields were scrutinized; and in the syntactic level thematic roles and nominalizations were examined.

In choosing the title ‘**Wal Agarraa**/the time will come/‘, the artist seems to threaten those who conspire on the Oromo and try to cover up their wrongdoings. The artist repeats his stance for siding with ‘truth’ by highlighting that those who committed wrong cannot get away with their deeds forever. The song commences with a splendid view of Lake Wanci whereby an empty Oromo chair known as ‘**barcuma’** was seen on the shore. The Oromo traditional chair, ‘**barcuma’**, often symbolizes power or authority. The empty ‘**barcuma’** probably implies that the Oromo people do not have a genuine leader at the moment. One may assume that the lake represents the Oromo people. It may also imply that the people need to have a leader.

The way it commences, there is an empty Oromo traditional seat known as ‘**barcuma’** seen on the flanks of Wanci Lake. In Oromo Gada System ‘**barcuma’** symbolizes power or authority. Towards the end of the song, a whip was placed on the ‘**barcumma’**. The whip symbolizes the justice system among the Oromo. The whip was placed on the **bracumma** at the end of the song. Therefore, the artist conveyed the message that someone who is morally upright to do justice shall preside over the people but for the moment, there appears to be no one good enough to fill the void. Therefore, only the whip was put on the ‘**barcumma’** to signify that at the moment the Oromo do not have true leaders. The artist holds a spear staring at a blank lake as if he were looking for the presumed leader beyond the lake.

Galanaa portrayed the Oromo as a great nation in the Horn of Africa. He attributes the greatness of the Oromo to the possession of a unique egalitarian democratic system of governance called the Gada System. The Gada system is associated with democratic principles whereby the ‘truth’ will be upheld in democratic processes. Accordingly, Gada is based on justice for all, humility in victory and use of democratic means to fight injustice. The verses elaborating this point are verses 39, 68, 69, 70 and 77. As such the artist implies that although there is immense pressure to suppress justice, truth will eventually be revealed. Particularly, verses 68–70 show that truth moves slowly but it will overshadow conspiracies once it is revealed.

Meanwhile, Galanaa appears to compare the situation of Oromo with the roasting of coffee. There is a girl who roasts coffee and apparently pounds it before she serves it to the people in a house. The pillar of the hut symbolises the greatness of the Oromo as evidenced by [16]**Guddaan gaanfa Afrikaa**/the great of Horn of Africa; [17]**Oromookoo mitiiree**/is it not Oromo. Galanaa seems to answer the question he posed earlier (what was the oath from the outset) by stating that the demands of the Oromo can't be hushed up and overlooked any more. Galanaa seems to reaffirm that the Oromo are the largest in the entire Horn of Africa. As a region, Oromia is the largest in Ethiopia. The Oromo also have got a very effective system called the Gadaa system for managing their resources and making transition of power democratically. It appears that Galanaa was perplexed by the paradox that despite all these, the Oromo remained under the influence of others as they are subjected to a host of abuses and mistreatments. So, the artist expresses the dilemma that a group as large and resourceful as Oromo could not decide its fate.

The moral lesson of the song was that the demands of the Oromo people in general and the Qeerros and Qarrees (Oromo male & female youths who were the major actors during the Oromo struggle) in particular have been betrayed. The song encouraged the Oromo to seek justice and remain morally upright and vigilant than being misled by temporary gains of those who have ill motives. The title of the song, **Wal Agarraa**/the time will come/foreshadows the inevitability of the revelation of the ‘truth’ of the Oromo. In other words, the artist is trying to threaten the injustices against the Oromo.

Birraa/spring symbolizes the period when people get relief from the challenges of the rainy season The Oromo believe that summer is with gloomy moment wherein mankind is faced with uncertainty. The rainy season symbolizes uncertainty, thunder; overflowing rivers and lack of transportation are subsumed. Galanaa foreshadows a bright time ahead for the expecting mass who are enduring the problem. The song has many instances of intertextuality of traditional Afan Oromo proverbs that have been inserted/alluded to it. It should be enhanced intertextuality -- one tool of critical speculations scanning text and discourse. Some of these are: sibilallee sibiilatu qaraa/it is only metal that can file knife/; dhugaan nama galagala galaa/justice will alwys be done eventually/; Supheen gaachana hin ta'u/as it can't be an armour; Leencca ta'e dhaladheen/I was born as a lion.

Birraa wal agarraa implies that a bright time awaits those seeking justice. The verses: yoo jabaannee birraa wal agarraa/if we could manage it, we will meet in the spring; yoo jirannee birraa wal agarraa/we shall see each other in the spring if we could make it/. Thus, Galanaa foretells that the Oromo will overcome the challenges that have piled up at the moment. To embolden the Oromo, Galanaa reiterates that the Oromo have already achieved their demands previously when he says, wal agarrees jirra/we have already seen each other/.

The lyrics of **Wal Agarraa** subsume elements of past glory, pride, betrayal and the aspiration to reclaim past glory. **Ijoollee biyya kootii gugurraa matan furaa eenyutu eenyun haale maal irbuun gaafa duraa** shows the betrayal and conspiracy made against the Oromo. The key stanza of the song**..yoo jabannee birra wal agarraa**/if we could manage it, we shall see each other in the spring/; **yoo jirannee birraa wal agarraa**/if we could manage it, we shall see each other in the spring/as the last stanza reflect the optimism of the artist that the justice of the Oromo will be served/truth will be revealed eventually.

[14] **Maaltu faarsa miciree**/no one will appreciate bushes
[15] **Guddaan gaanfa Afrikaa**/the giant of Horn of Africa
[16] **Oromookoo mitiiree**?/is it not my Oromo?
[17] **Oromiyaa mitiiree?**/is it not Oromia?
[18] **Sirna gadaa mitiiree?**/is it not Gadaa System?
[19] **Oromummaa mitiiree?**/is it not Oromumma?
[20] **Gadaa fi dhugaadha kallachi keenya** (x2)/Gadaa and truth are our emblem
[21] **Jifuu fi safuudha kabajni keenya** (x2)/norms and ethics are our principles
[22] **Gadaa malee hin faarsinu gadoo**/we appraise Gadaa; not misery

The verses from 14-22 reflect the past glory that the Oromo want to reinvigorate. Particularly, verse 20 which holds that the Oromo want to be guided by Gada and truth which are epitoms of the Oromo egalitarian system.

In the opening verse, **Nan geerara aarii koo**/**I will express my anger through ‘gerarsa’**, the artist justifies why he sang the song. ‘Gerarsa’ is a popular Oromo music which is often used to express resentment and anguish. The artist outlines the reason behind the song lyrics-that he wants to express his fury which is given in the second verse, **kan na keessaa boba'u/the fury that is burning in my heart**. A fury burning implies that the level of anger is immense. He uses metaphors, coward and lion in verses 5 and 6; **Leencca ta'e dhaladheen**/**I was born as a lion**; **kodhoollemmoo ta'a ree**/**how could I be a coward**. He seems to suggest that he will not bow down but rather he will remain vigilant in the face of threats. This is shown in verses 7 and 8 (**namni bakakkaa beeku**/**he who is accustomed to the sound of** thunder; **dibbefimmo nannahaa ree**?/**does one shake when drums**^2^ **are** beaten?) wherein he states that he had the experience to manage tough situations as he underscored that he does not fear the sound of thunder let alone startle upon hearing the sound of a drum.

Galanaa addresses the Oromo youths (‘**Gugurraa mataan furaa/black people with plaited hair**’). The qeerroo and qarree used to grow their hair and get it plaited to signify their status during the Oromo Protests. He poses a question to the qeerro and qarree regarding broken promises (**Eenyutu eenyuun haale/who has betrayed whom**?). He was referring to a solemn oath that has been denied. As such, he was implying certain ‘others’ have betrayed the qeerroo and qarree and implicitly the Oromo. However, the artist has identified himself among those seeking truth. He noted that the Oromo abide by moral principles (**Gadaa fi dhugaadha kallachi keenya**/**Gadaa and truth are our emblem**/**Jifuu fi safuudha kabajni keenya**/**norms and ethics** are our principles). Implicitly, the artist was referring to groups that have conspired against the rights of the Oromo. He underscored that the wrongdoers cannot get expiation for they lack the wisdom of repentance. (**Fala hin argatu namni faloo hin beekne/he who doesn't have wisdom can't get expiation**).

The artist also presents his determination to stand firm against any threat from the powerful group (**Na nyaartulle hinnawu gonkaa/even if you frown at me**; I won't be frightened; **Dhugaa irraa maaltu na dhorkaa/who can prohibit me from pursuing truth**). He intoned that those seeking truth will be winners eventually. However, wrongdoers will be ashamed of their evil acts (**Eessatti kufee namni dhugaaf duulee/what has befallen he who upheld truth; Galgalli hintoluu namni sobaaf duulee**/the destinies of those who pursue falsehood are futile).

Furthermore, the artist expresses the magnitude of the conspiracies of the opponents using a common proverb in Afan Oromo. There is a popular proverb used by the Oromo as they preach systems of peaceful coexistence. It holds that one has to try different mechanisms to help persons with ill motives to refrain from wrongdoing. As such, some errors should be tolerated, some errors should be hidden and some others should be used in the form of advice. (**Egaa ergan kudhan obsee**/I **tolerated some errors** ten times; kudhan dhoksee **kudhan numan gorsee/I hid of some errors; I gave him council for some errors**). But the artist has outlined what should follow should the wrongdoer fail to refrain from his errors amid these attempts. He opined that patience has got limits. He underscored that although he bears **bokku/symbol of power**/to seek peaceful solution, the same hands can also bear the spear-signifying that he can fight the perpetrators after all peaceful mechanisms failed. (**Harkii bokkuu qabeebodees numa qabaa**/the same hand that holds **bokku** (sceptre; symbol of power) **can also bear a spear**).

The artist forecasts an eminent threat in verses 56–59 wherein he reiterates that what they have speculated has materialised for the second time. This implies that such has been a trend-that the Oromo have been betrayed on different occasions. The artist also expresses his dismay that he has hidden evil acts of perpetrators **Obsuufi dhoksuu koo dabuma seetee?**/I concealed your errors but you took my patience as cowardice/. Galanaa threatens the evil doer that he cannot rest assured that the evil act has not been revealed for the moment. But the artist noted that the wrongdoer shall not take the hiding as an act of cowardice. The artist is instigating the Oromo to stay firm as they face the perpetrators. The song is meant to ascertain that justice will be done-**dhugaan namaa galagala gala/justice will always be done** eventually which is repeatedly shown in verses 27–29, 77–79 and 91 among others.

Galanaa seems to threaten those who assassinated Haacaaluu Hundessa, a popular Oromo artist. The artist vows to pursue truth whatever the consequences may be. He further notes that death is inevitable and he doesn't fancy life after Haacaaluu's death (verse 87) and life shall be lost for a hero (verse 88). One could see that the artist is threatening wrongdoers as he repeats the verses (**Yoo jabaanne, birraa wal agarra/if we could manage it, we shall see each other in the spring; yoo wal taane, moonee wal agarra** (x2)/if we are united, we shall see each other after victory; **Wal Agarra garraa, wal agarraa garraa(x2)/we shall see each other; we shall see each other**.

## 3 Pragmatic level

At the pragmatic level, we identified speech acts and the uses of pronouns that indicate the relationships and roles within the discourse. These elements, in turn, were linked to the political discourse strategic functions to achieve a deeper understanding of the ideas and motivations underlying this discourse.

### 3.1 Pronouns

There are pronouns that represent the linguistic socio-political relations that existed among the actors in this particular discourse. The pronouns and their different variants assign roles and define spaces among those involved in the discourse. The participants included the social group with whom the artist identified himself, those whom he opposed by alluding them with wrongdoings, and to whom the discourse was directed among others.

To represent the morally upright inner group, the artist has used the first person pronouns. This can be evidenced in verses [9]**ijoollee biyya kootii**/**my fellow** countrymen; [20**]Gadaa fi dhugaadha kallachi keenya**/Gadaa and truth are **our** emblem; [21**]Jifuu fi safuudha kabajni keenya**/norms and ethics are **our** principles; [22]**Gadaa malee hin faarsinu gadoo**/**we** appraise Gadaa; not misery; [23]**Lubaan bullaahin qabnu madoo**/**we** want to be led by elites; not the wicked; [50]Yoo **jabaanne**, birraa wal agarra/if **we** could manage it, we shall see each other in the spring; [51]**Yoo wal taane**, **moonee wal agarra**/if **we are united**, we shall see **each other** after victory; [52]**Wal agarra garraa, Wal agarraa garraa**/**we** shall see **each other**; **we shall see each other**; [53]**Wal agarrees jirra**/**we have seen** each other [54]Walis **arguuf jirra**/**we are to see each other**; [55]**Wal agarra**/**we shall see** each other.

[56]**Birraa wal agarraa/we shall see** each other in the spring [57]**Yaa ijoollee…yaa ijoollee…/my people**…my people [58]**kan jenne hin oollee** “**nui**”/what **we** speculated has materialised, [59]**Yaa ijoollee**…**yaa ijoollee**…/my fellows…my fellows; [60]**ammas nafoolee**/it has beset me “**na”/**me; [61]**Gadaa malee hin faarsinu gadoo**/**we** appraise Gadaa; not misery and [62]**Lubaan bullaa hin qabnu madoo**/**we** want to be led by elites; not the wicked; that helped to legitimize his position and the social group with whom he identified himself.

These verses help to identify the speaker as a member of the group that is seeking justice. In these verses, both first person singular pronoun and the first person plural pronoun also grouped the speaker and the social group with whom he identified - in this case, the Oromo seeking truth to be known and justice to be served. This self-inclusion implies the legitimization of the demands of the Oromo and vindicated its position within the social framework.

On the contrary to portray the morally wicked group and the inevitability for truth to be revealed at the eventual demise of the perpetrators, the artist used second person pronoun **‘sii’/you** and third person pronoun **‘isa’/he/**in verses [24]Nan gala **hin jedhu namni osoo hin geenye**/no one will return before he's reached his destination; [25]**Fala hin argatu namni faloo hin beekne**/**he who doesn't have** wisdom can't get expiation; [35]Natu **sii baataa Siituu of dadhabaa**/I catered/cared for **you** tirelessly but **you** despised my catering [36]**Obsuufi dhoksuu koo Dabuma seetee**?//I covered up **your** errors; but **you** contemned my effort; [37]**Ni beektii garaan koo Akka na gootee**'**ati**’/I knew instinctively what **you** have done against me; [44]**Eenyutu na'aaf bubbuta nyaara** ‘**ati**’/no one will startle when **you** frown [45]**Keessi hin mul'atu beeki garaan dhiiraa**/make no mistake (**you**); no one knows what is there in a hero's heart [46]**Maaf na nyaaraa (inni) na nyaaru kanarra**/why does **he** frown at me; **he** can't frown at me anymore [63]**Nan gala hin jedhu namni osoo hin geenye**/no one will return before **he's** reached his destination; [64]**Fala hin argatu namni faloo hin beekne**/he who doesn't have wisdom can't get expiation [65]**Nanyaartulle hinnawu gonkaa**/even if **you** frown at me; I won't be frightened [83]**Eenyutu na'aaf bubbutta nyaara**/no one will startle when **you** frown; [84]**Keessi hin mul'atu beeki garaan dhiiraa**/make no mistake; no one knows what is there in a hero's heart.

So it could be seen that in reference to the powerful group, the singer used **‘isa’ ‘he** ‘and **‘sii’ ‘you** ‘which clearly marked a distance from the inner group and hence different roles. In verses where the third person singular pronouns were used, the powerful groups were questioned. Furthermore, in other verses, the second person pronoun was used in reference to the perpetrators. In short, all uses of pronouns referring to those in power were directly related to the strategic role of delegitimization of the discourse of the powerful and legitimization of the rebellion of the dominated.

As shown above, the use of pronouns assigned social roles and relationships that could be associated with the strategic function of legitimization-delegitimization. However, the imposition of such roles within the discourse also involved the function of coercion, since almost arbitrarily; the different actors have been placed in certain spaces within the social framework wherein the powerful group attempted to threaten the rebellious.

### 3.2 Speech acts

The theory of speech acts is not confined to individual words or sentences that form the basic elements of communication. It is rather about particular speech acts that are performed when uttering words that explicate what people do when they speak (Fairclough 2001). Thus, speech act is the attempt to get things done merely by speaking. Instances of speech acts are abound in the selected song.

### 3.3 Assertive speech acts

Assertive speech acts were used in the fragment and acquired consistency through true propositions expressed in verses [1–8]. Through these propositions, the artist intended to reassure himself as the enlightened person regarding the injustice being perpetrated against the Oromo and he assumed the role of informing the Oromo about the abuses. The one in verse [5–8] Leencca ta'e dhaladheen/I was born as a lion; [6] **kodhoollemmoo ta'a ree**/how could I be a coward; [7]**namni bakakkaa beeku**/he who is accustomed to the sound of thunder; [8]**dibbefimmo nannahaa ree?/**doesn't shake when drums are hit show the commitment of the artist to fight injustice at any rate.

The strategic function of legitimization-delegitimization was associated with these speech acts. The artist legitimized his discourse from the resistance point of view while he delegitimized the threats generated by the powerful as they attempt to perpetuate the conditions the status quo. As such this discourse delegitimized the threat by the powerful to suppress the Oromo. He instigated the need to fight the injustice at any rate.

The artist has instigated that justice may be delayed but it cannot be hushed up forever. Verses [69–71] in particular support this notion. The artist has equated truth with a mountain so as to show the inevitability that reality will defeat all hurdles to shine eventually. These verses portray truth as something that illuminates eventually by defeating all the challenges; [69]**Dhugaan qaxxisa tiratti**/**truth** moves slowly; [70]**Akka arge hin deemtu karaadha filatti**/truth does not fan out; **it travels on selected paths**; [71]**Gaafa olbaatu tulluudhaan mul'atti**/upon reaching the **top it is observed like a hill**; [72]**Gowwaan hosseen karra cufa**/the foolish shuts a door using hollow barks; [73]**Namni kufuu hawwe teessumatti kufa**/one that opts to fail would fall from the seat; [79]**Gara kuteessatti dukkanni ni ifa**/but to the brave, darkness will glow.

These directives or assertive speech acts correspond with coercive strategic functions; i.e., they involved some degree of control over the actions of the person receiving the discourse or to the ones questioned through it. These are [46]**Maaf na nyaaraa na nyaaru kanarra**/**why does he frown** at me; he can't frown at me anymore; [47]**Maalan aarasi hin aaru kanarra**/**why do I get angry**; I won't be riled anymore; [48]**Maalan boo'a hin boo'u kanarra**/**why do I weep**; I won't weep anymore; [49]**Yoo jiraanne, birraa wal agarra** (x2)/we **shall see each other in the spring** if we could make it/which also correspond with the strategic function of coercion.

### 3.4 Semantic level

The song was released against the backdrop of a period when the Oromo people have been immersed into a deep sense of anger and anguish following the coldblooded murder of Haacaaluu Hundessa, a famous Oromo artist and the subsequent crackdown on prominent Oromo politicians. Galanaa has expressed his resentment about the assassination of his compatriot who was his inmate artistically as evidenced by the following verses: [87]**Gaafa giitii lubbuun haa dabartu**/on the eventful day may life be lost; [88]**Eega Haacaaluu lubbuun nan marartu/**I don't fancy life after Haacaaluu's death.

The first verses of the song are in the form of gerarsa (type of song for expressing lamentation and resentment among the Oromo most often). Gerarsa is often presumed to be a kind of song used to express the injustice. It appears that Galanaa used the geressa deliberately to express the resentment of the Oromo. The artist expressed his determination to pursue justice using the following stanza: [1]**Nan geerara aarii koo**/I will express my anger through ‘gerarsa’; [2]**kan na keessaa boba'u**/the fury that is burning in my heart/.

Furthermore, the artist instigated the need to pursue truth at the lexico-semantic field. Thus, the artist resisted injustice implied endeavouring to counter the inequality produced, reproduced and legitimized by the powerful group. The semantic field under consideration is expressed in verses [26]**Na nyaartulle hinnawu gonkaa**/even if you frown at me; **I won't be frightened** in which the speaker began legitimizing his particular version of *seeking truth*. The artist had delved into the action in verses [27]**Dhugaa irraa maaltu na dhorkaa**/no one can prohibit me from pursuing the truth; [28]**Eessatti kufee namni dhugaaf duulee**/what has **befallen he who upheld the truth**; [29]**Galgalli hintoluu namni sobaaf duulee/the destiny of one who pursues falsehood is futile**.

The Oromo believe that **dhugaa/truth** is the son of the creator and by transitivity anyone who denies truth is fundamentally in collision terms with the creator. **Jifuu fi saffudha** ... **keenya/**. **Safuu** ‘ethics’ is the system through which the Oromo distinguish between right and wrong. As per this parable, to lie is **safuu.** There are several relevant lexico-semantic elements in verses [26], [27] [28] and [29] which allow to visualize the sophisticated articulation of a system that is based on falsehood which is destined to fail more clearly.

The words highlighted in bold type above correspond to a semantic network in close relation to the institutional violence that mobilized the content and argument of this discourse. As such, these linguistic choices reflect the existence of a social order established and maintained by the powerful to perpetuate a system of leadership that only benefits the powerful entities while undermining the right of the Oromo which was sternly criticized and opposed by the resistance song.

### 3.5 Syntactic level

In this song, the artist appears to be referring to the Oromo protest wherein the qeerro and qarree (Oromo male and female youth respe-ctively) were the proponents in ousting the regime in Ethiopia in a popular struggle termed as Oromo Protests. The verse **Gugurraa mataan furaa/black people with plaited hair [14]*,*** refers to the hairstyles of the Oromo youths (qeerroo and qarree) during the Oromo Protest. The youth used to grow and braid their hair during the Oromo protests. The central theme of this stanza is that the qeerroo and qarree who played the pivotal role in ousting the previous regime could not reap the fruits of their struggle because the struggle was hijacked which is evidenced by verses 11–19 ***Eenyutu eenyuun haale/who has betrayed whom;*** whereby the artist reminded his audience about an oath of allegiance that was not accounted for in verse [12];**Maal irbuun gaafa duraa**/what was the oath from the outset? [13]**Osoo birbirsi jiru**/in the presence of ‘birbirsa-podocarpus falcatus’ [14]**Maaltu faarsa miciree**/no one will appreciate bushes. There is symbolism in here whereby the artist represents the role of the Oromo youth with ‘**birbirsa’** podocarpus falcatus that signifies huge achievements among the Oromo. The artist seems to suggest that those reaping the fruits of the Oromo struggle are those whose roles were insignificant (bushes).

These stanzas seem to suggest the great deal of resentment among the Oromo people following the derailing of the trajectory of the reform. The artist was criticising the lack of respect for the rights of the Oromo and **Oromumma** (the sense of being Oromo) in particular the verse ***Maal irbuun gaafa duraa/what was the oath from the outset.*** He tried to indicate that there were promises made for the Oromo people but the authorities failed to uphold their rights at the moment. The artist seems to suggest that the Oromo are reeling with anger and resentment as symbolized by the coffee roasted and pounded before it was served in the musical clip.

However the artist also upholds that the Oromo cause is led by the principles of the Oromo wisdom reflected in the Gadaa system and hence it can't be pushed aside by any force. Gadaa is a great system seen by many as rivalling democracy of Greece that the Oromo contributed to the world as signified by verses 15–19. [15]**Guddaan gaanfa Afrikaa/the giant of Horn of** Africa [16]**Oromookoo mitiiree**?/is it not my Oromo? [17]**Oromiyaa mitiiree**?/is it not Oromia? [18]**Sirna gadaa mitiiree**?/is it not Gadaa System? [19]**Oromummaa mitiiree**??/is it not Oromumma?

In the song, **faloo** refers to the process of expatiation whereby someone who has trespassed can be cleansed from his wrongdoings. This is a common process among the Oromo whereby trespassers will confess their crimes in **jaarsumma**^3^. The guilty party will settle the problem through mediation so as to curb the escalation of repentance. Galanaa seems to suggest that the authorities should address the demands of the Oromo in order to sustain peace in the country. The part of a slaughtered animal which was hanging on the fence symbolizes the way the Oromo expatiate their trespassing. It is believed that the **Qallus** (spiritual leaders of the Oromo) will foretell the previous, present and future courses of events.

The artist implied that the authorities should not rest assured considering the silence of the public. He reiterates that the silence does not signify the indifference of the Oromo to injustices being perpetrated. This can be observed by deciphering the verse, **sangaan usnaan iyyite qacceen** [81]/the whip shout louder though the ox keeps silent; which shows that the silence of the victim in response to abuses perpetrated does not signify normalcy; the victim can take any measure on the right time for no one could understand what lies in the heart of braves. This claim is further established in [84] **keessi hin mul'atuu beeki garaan dhiiraa/**makes no mistake; no one knows what is there in a hero's heart.

However, in the following stanza the artist speculates that the time of mistreatments and abuses won't last long and truth shall remain the winner eventually. He signifies the dynamism of the events; the artist encourages the victims by using the following stanzas. Thus, Galanaa encourages the public by insisting on dynamism indicating that the current status cannot last forever. He foreshadows that we shall meet in the spring having defeated the enemy in verses:

In the following verses the artist takes his audience back to reflect the fear he predicted that groups that are against the Oromo have seized power and miseries are being perpetrated on the public. The powerful group is reaping the fruits of the Oromo struggle. [57]Yaa **ijoollee…yaa ijoollee…/my fellows…**my fellows [58]**kan jenne hin oolle(x2)/the speculated has materialised**; [59]**Yaa ijoollee…yaa ijoollee**…/my fellows…**my fellows**; [60] **Ammas na foolee** (2)/**once again it has beset me**. The artist encourages the Oromo to intensify their struggle to overcome the imposition of the powerful.

## 4 Conclusion

The objective of this work was to propound an Oromo protest song through the textual analysis of the political instrumentality of its lyrics so as to enunciate the Oromo resistance. As such, the song encouraged the Oromo to vehemently struggle for justice through many underpinning themes. As Oromos, we had witnessed the suppression of the Oromo demand for their rights. To this end, we delved into analyzing the song as a form of political discourse using critical discourse analysis, CDA.

In this study, we have attempted to demonstrate that **Wal Agarraa**, an Oromo resistance song, was a protest art that spoke on behalf of the Oromo people by pointing out the injustices and malpractices they faced. We have tried to enunciate that the selected protest music assisted in the interrogation of the society's moral sphere. As such we argued that this music as a kind of text conveyed discourses that are partly shaping emotion of the Oromo as they experience injustices. Thus, we believe that the song was not merely a frivolous component of various sects of Oromo culture, or passing sources of insignificant entertainment. Instead, the song and the singer reflect the experience of the Oromo in resisting any sort of domination during specific period. The artist played his role to portray the passions of the Oromo people as he spurred collective mind sets of resistance across many social aggregates through his appeal to their desires, their morals, their lamentations and their angers.

## Declarations

### Author contribution statement

Kassaye Gutema Adama and Alemgena Belete Abdeta: Analyzed and interpreted the data; Wrote the paper.

### Funding statement

This research did not receive any specific grant from funding agencies in the public, commercial, or not-for-profit sectors.

### Data availability statement

No data was used for the research described in the article.

### Declaration of interests statement

The authors declare no conflict of interest.

### Additional information

No additional information is available for this paper.

## Footnotes

1 The Oromo Protest was a popular movement by the Oromo (the largest ethnic group in Ethiopia) in response to the political domination. It started when the then government introduced a controversial master plan to extend the capital of Ethiopia into Oromia regional state. The Oromo Protests forced the government to cancel the master plan but the struggle raged until the then prime minister resigned.

2 Drums are beaten (metaphor)- he who is accustomed to sound of thunder; does one shake when drums are beaten-these verses imply that those who are fighting the injustice against the Oromo are brave and that they have got experience; they are not cowards who shake for simple things.

3 Jaarsumma-is a traditional fact finding process commonly used to settle disputes among the Oromo. It is a process which helps to maintain peaceful coexistence of the society.
